# Supplementary material for: Modelling the drivers of outbreak communication in online media news for improved event-based surveillance
Source: PLoS One. 2025 Aug 4;20(8):e0327798. doi: 10.1371/journal.pone.0327798 (PMC12321081; doi:10.1371/journal.pone.0327798)

Supplementary file 1.

A bipartite network of sources communicating on outbreaks between 2018 and 2019 extracted with the PADI-web tool

A. Avian influenza

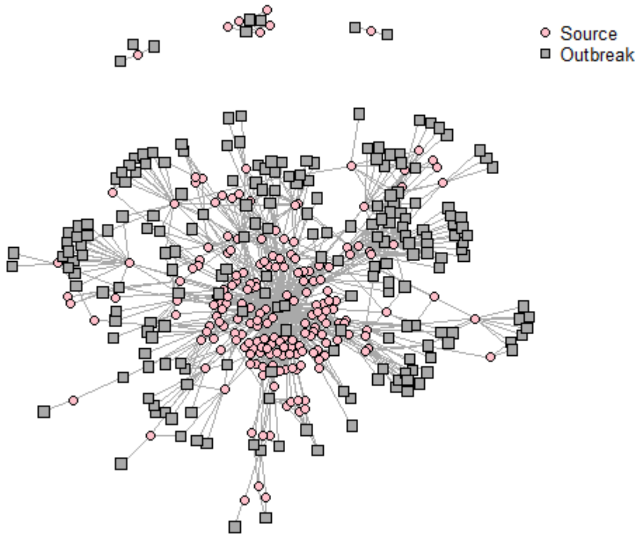

B. African swine fever

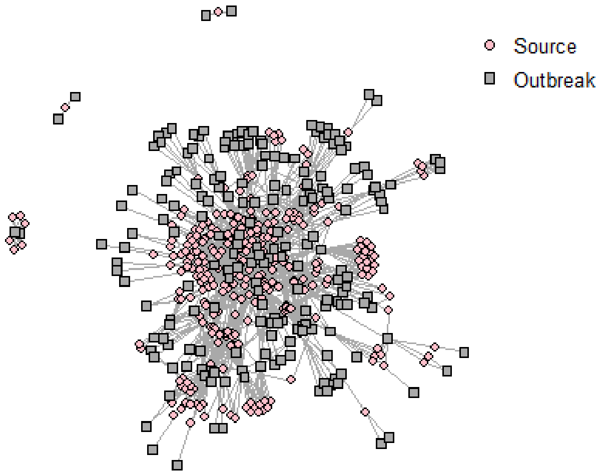

Supplement: S1 File — (PDF) [file pone.0327798.s001.pdf]
