# Supplementary material for: Modelling the drivers of outbreak communication in online media news for improved event-based surveillance
Source: PLoS One. 2025 Aug 4;20(8):e0327798. doi: 10.1371/journal.pone.0327798 (PMC12321081; doi:10.1371/journal.pone.0327798)

Supplementary file 2.

Degree distribution for the node sources and node outbreaks of the bipartite networks

A. Avian influenza

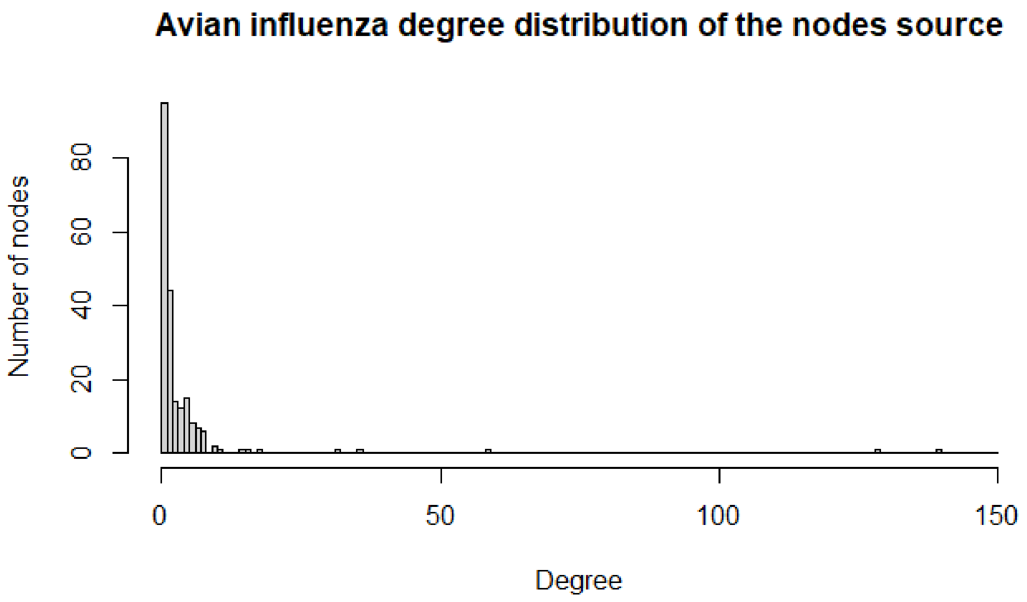

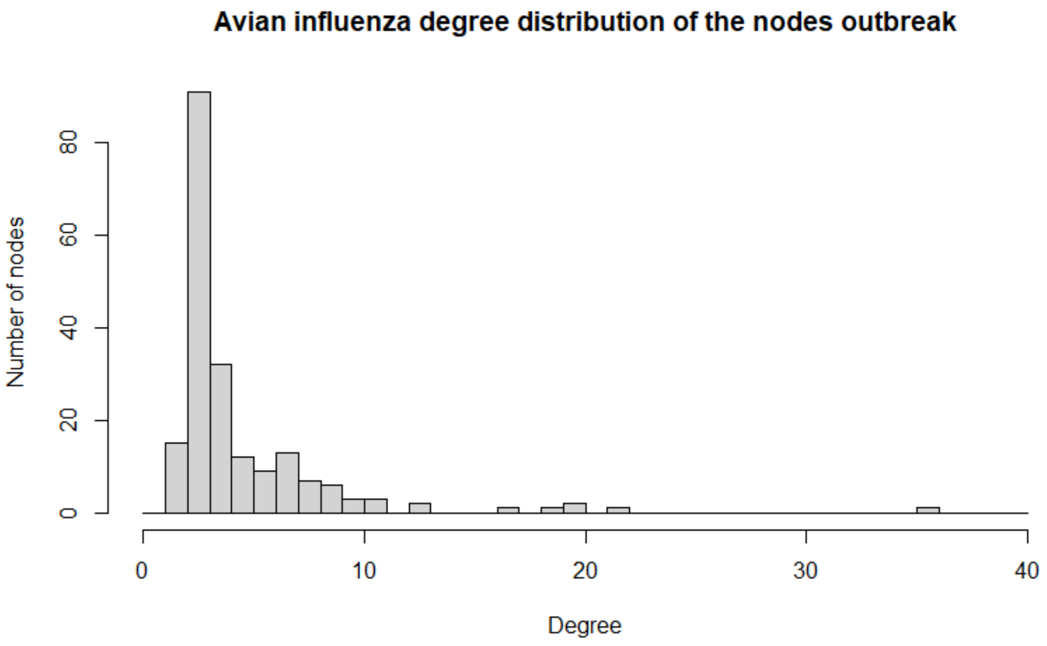

B. African swine fever

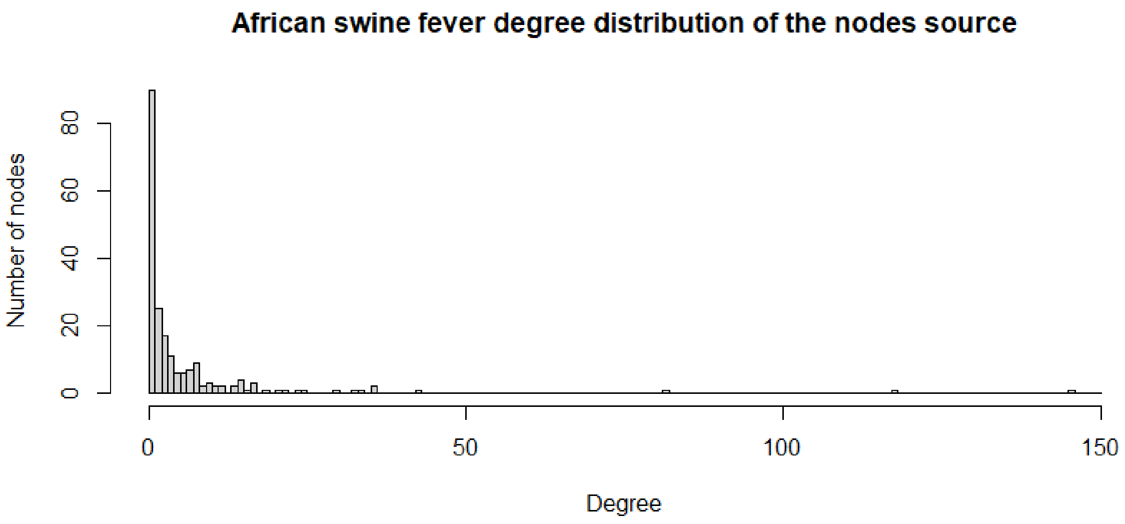

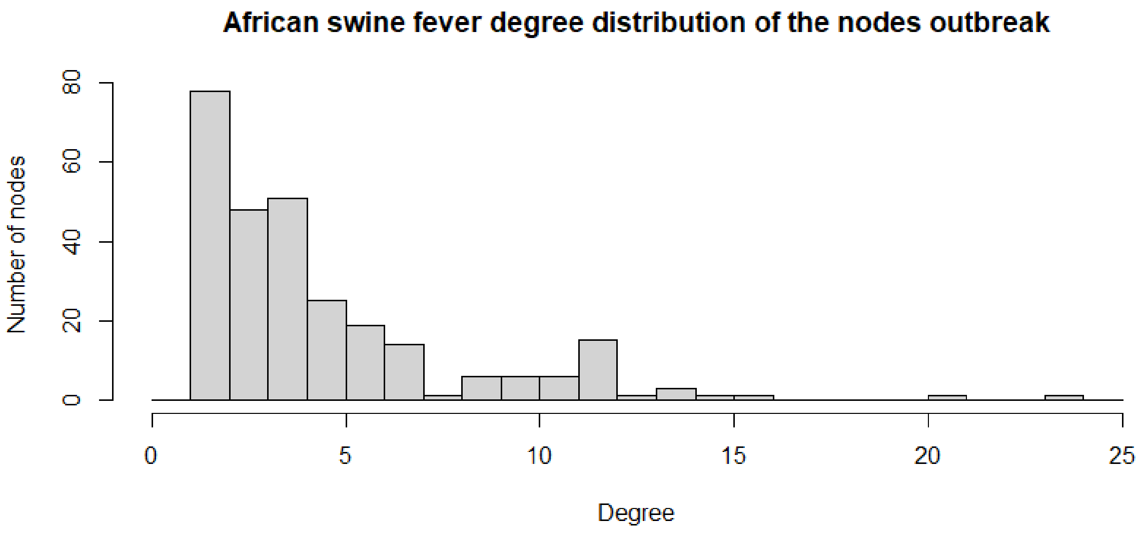

Supplement: S2 File — (PDF) [file pone.0327798.s002.pdf]
