# Supplementary material for: Modelling the drivers of outbreak communication in online media news for improved event-based surveillance
Source: PLoS One. 2025 Aug 4;20(8):e0327798. doi: 10.1371/journal.pone.0327798 (PMC12321081; doi:10.1371/journal.pone.0327798)

Supplementary file 3

A. In-sample performance for the Exponential random graph Models (ERGM) selected for **Avian influenza**, by a goodness-of-fit (Gof) analysis comparing three structural statistics of the observed network to those of networks randomly simulated by the adjusted ERGM (distribution of degree per type of node and minimum geodesic distance).

a) Gof for the degree of outbreak nodes

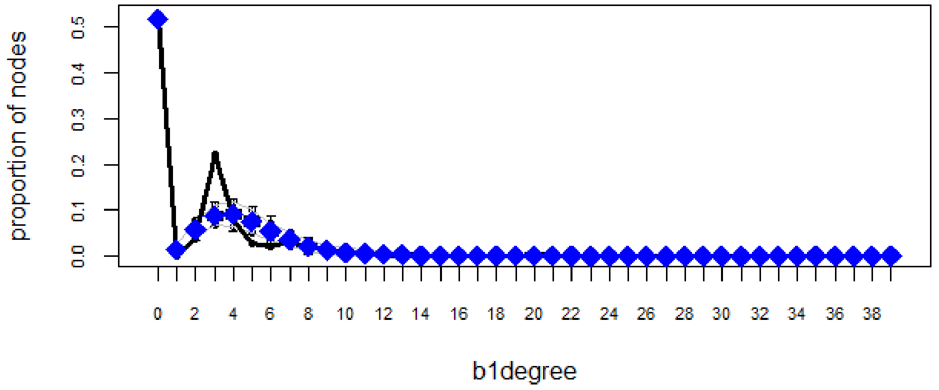

b) Gof for the degree of source nodes

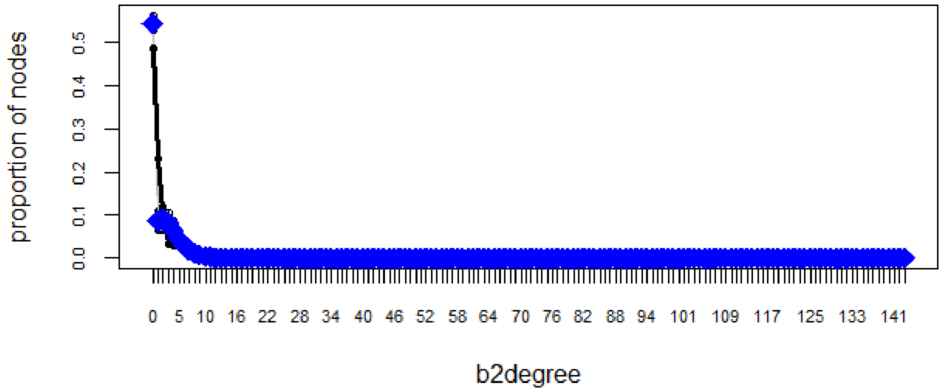

c) Gof for the minimum geodesic distance

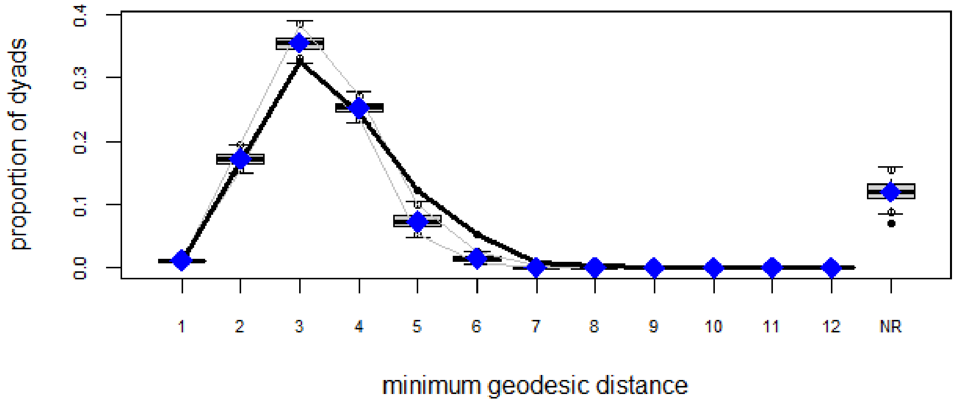

B. In-sample performance for the Exponential random graph Models (ERGM) selected for **African swine fever**, by a goodness-of-fit (Gof) analysis comparing three structural statistics of the observed network to those of networks randomly simulated by the adjusted ERGM (distribution of degree per type of node and minimum geodesic distance).

a) Gof for the degree of outbreak nodes

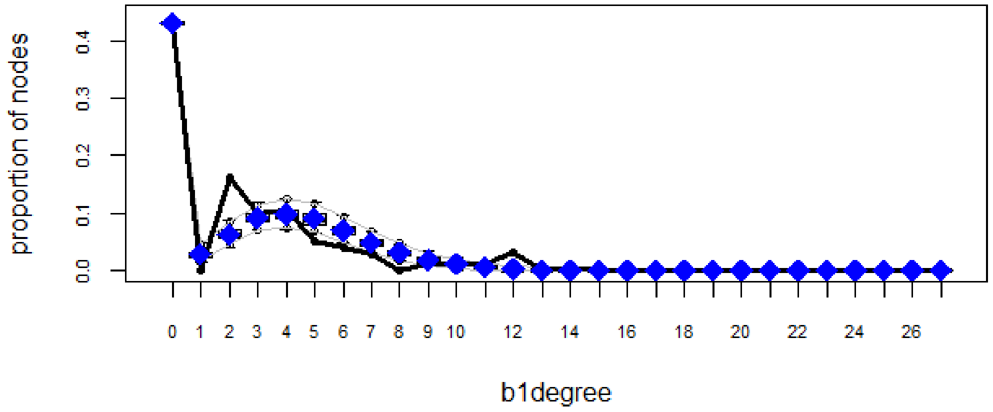

b) Gof for the degree of source nodes

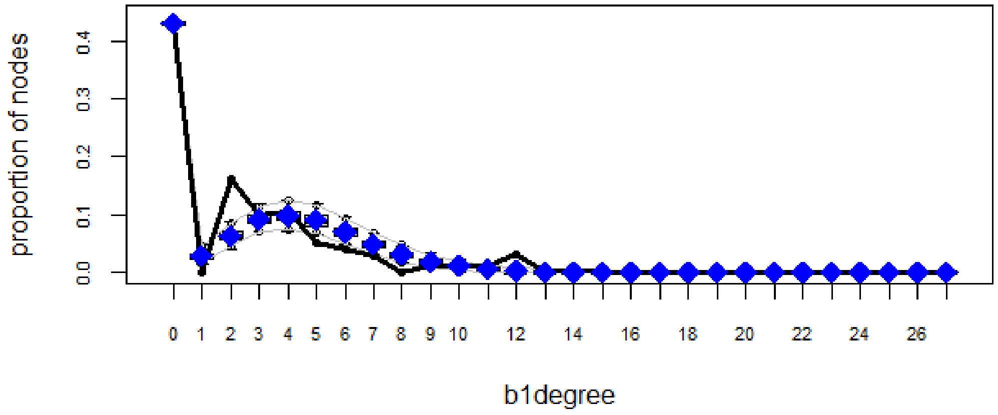

c) Gof for the minimum geodesic distance

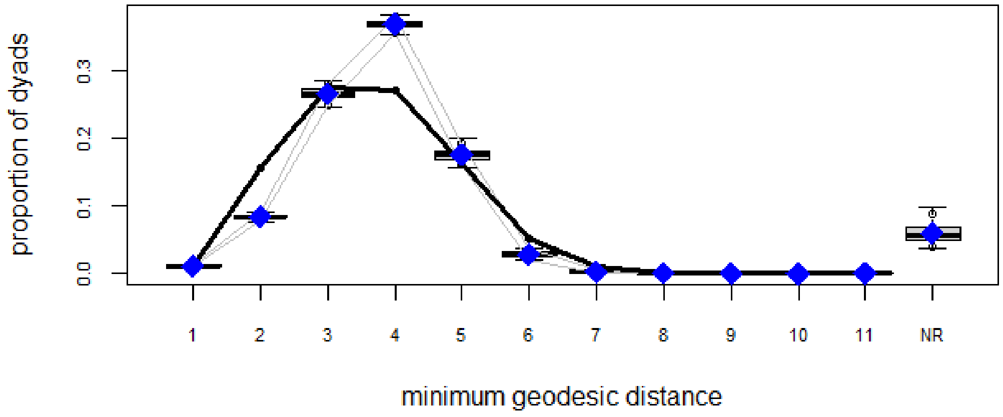

Supplement: S3 File — (PDF) [file pone.0327798.s003.pdf]
