# Supplementary material for: Modelling the drivers of outbreak communication in online media news for improved event-based surveillance
Source: PLoS One. 2025 Aug 4;20(8):e0327798. doi: 10.1371/journal.pone.0327798 (PMC12321081; doi:10.1371/journal.pone.0327798)
Supplement: Attachment — Submitted filename: PONE-D-24-25259 (003)_16Aug24_comments.pdf [file pone.0327798.s004.pdf]

PLOS ONE

Modelling the drivers of outbreak communication from event-based surveillance for improved epidemic intelligence

--Manuscript Draft--

**Manuscript Number:**	PONE-D-24-25259
**Article Type:**	Research Article
**Full Title:**	Modelling the drivers of outbreak communication from event-based surveillance for improved epidemic intelligence
**Short Title:**	Drivers outbreak communication online media
**Corresponding Author:**	Elena Arsevska<br>CIRAD Montpellier-Occitanie<br>FRANCE
**Keywords:**	drivers, outbreak communication, online media, event-based surveillance, exponential random graph model, bipartite network, network modelling
**Abstract:**	Epidemic intelligence (EI) practitioners at health agencies monitor various media sources to detect and follow up on disease outbreaks. Several tools, such as PADI-web (Platform for Automated Extraction of Disease Information from the Web), automatically monitor and filter outbreak-related news on the web, facilitating the risk assessment by EI practitioners. However, in the mass of collected news, there is a need to understand better what drives the communication on outbreaks by the different sources to allow for a more targeted and efficient EI process.<br>We built a bipartite network of sources communicating on outbreaks of avian influenza (AI) and African swine fever (ASF) captured by the PADI-web tool between 2018 and 2019 worldwide to assess a range of epidemiological, socio-economic, and cultural factors that drive communication on disease outbreaks.<br>Our AI network comprised 969 communicated news (links) from 436 news reports from 212 sources describing 199 AI outbreaks. The ASF network comprised 1340 communicated news (links) from 594 news reports from 204 sources and 277 ASF outbreaks.<br>An Exponential Random Graph Model (ERGM) was fitted for each network. In both models, international organisations and press agency sites were more likely to communicate about outbreaks than online news sites (OR=4.8 and OR=3.2, p<0.001 for AI; OR=3.1 and OR=4.7, p<0.001 for ASF). Research organisations for AI (OR=2.3, p<0.001) and veterinary authorities for ASF (OR=3.6, p<0.001) were also more likely to be a source of information than online news sites.<br>Our work provides insight into the factors driving online communication about animal and zoonotic infectious disease outbreaks. This information can guide EI practitioners to target specific sources based on their specialisation and global coverage and focus on certain world regions based on the disease's epidemiological status.
**Order of Authors:**	Solene Rodde
	Pachka Hammami
	Asma Mesdour
	Sarah Valentin
	Bahdja Boudoua
	Paolo Tizzani
	Lina Awada
	Carlene Trevennec
	Paulo Pimenta
	Andrea Apolloni
	Elena Arsevska

Powered by Editorial Manager® and ProduXion Manager® from Aries Systems Corporation

Additional Information:	
**Question**	**Response**
**Financial Disclosure**<br><br>Enter a financial disclosure statement that describes the sources of funding for the work included in this submission. Review the submission guidelines for detailed requirements. View published research articles from *PLOS ONE* for specific examples.<br><br>This statement is required for submission and **will appear in the published article** if the submission is accepted. Please make sure it is accurate.<br><br>**Funded studies**<br>Enter a statement with the following details:<br>• Initials of the authors who received each award<br>• Grant numbers awarded to each author<br>• The full name of each funder<br>• URL of each funder website<br>• Did the sponsors or funders play any role in the study design, data collection and analysis, decision to publish, or preparation of the manuscript?<br><br>Did you receive funding for this work?	Yes
Please add funding details.<br>as follow-up to "**Financial Disclosure**<br><br>Enter a financial disclosure statement that describes the sources of funding for the work included in this submission. Review the submission guidelines for detailed requirements. View published research articles from *PLOS ONE* for specific examples.<br><br>This statement is required for submission and **will appear in the published article** if the submission is accepted. Please make sure it is accurate.	H2020 MOOD project Grant agreement ID: 874850:<br>https://cordis.europa.eu/project/id/874850

**Funded studies**<br>Enter a statement with the following details:<br>• Initials of the authors who received each award<br>• Grant numbers awarded to each author<br>• The full name of each funder<br>• URL of each funder website<br>• Did the sponsors or funders play any role in the study design, data collection and analysis, decision to publish, or preparation of the manuscript?<br><br>Did you receive funding for this work?"	
Please select the country of your main research funder (please select carefully as in some cases this is used in fee calculation).<br>as follow-up to "**Financial Disclosure**<br><br>Enter a financial disclosure statement that describes the sources of funding for the work included in this submission. Review the submission guidelines for detailed requirements. View published research articles from *PLOS ONE* for specific examples.<br><br>This statement is required for submission and **will appear in the published article** if the submission is accepted. Please make sure it is accurate.<br><br>**Funded studies**<br>Enter a statement with the following details:<br>• Initials of the authors who received each award<br>• Grant numbers awarded to each author<br>• The full name of each funder<br>• URL of each funder website<br>• Did the sponsors or funders play any role in the study design, data collection and analysis, decision to publish, or preparation of the manuscript?<br><br>Did you receive funding for this work?"	BELGIUM - BE
**Competing Interests**	The authors have declared that no competing interests exist.

Use the instructions below to enter a competing interest statement for this submission. On behalf of all authors, disclose any competing interests that could be perceived to bias this work—acknowledging all financial support and any other relevant financial or non-financial competing interests.<br><br>This statement is required for submission and **will appear in the published article** if the submission is accepted. Please make sure it is accurate and that any funding sources listed in your Funding Information later in the submission form are also declared in your Financial Disclosure statement.<br><br>View published research articles from *PLOS ONE* for specific examples.<br><br>**NO authors have competing interests**<br><br>Enter: *The authors have declared that no competing interests exist.*<br><br>**Authors with competing interests**<br><br>Enter competing interest details beginning with this statement:<br><br>*I have read the journal's policy and the authors of this manuscript have the following competing interests: [insert competing interests here]*<br><br>* typeset	
**Ethics Statement**<br><br>Enter an ethics statement for this submission. This statement is required if the study involved:<br><br>• Human participants<br>• Human specimens or tissue<br>• Vertebrate animals or cephalopods<br>• Vertebrate embryos or tissues	Ethical approval was not required for this study as it includes information (news) freely available in the public domain. This research does not contain any studies with animals or humans performed by any of the authors.

Powered by Editorial Manager® and ProduXion Manager® from Aries Systems Corporation

• Field research<br><br>Write "N/A" if the submission does not require an ethics statement.<br><br>General guidance is provided below. Consult the submission guidelines for detailed instructions. **Make sure that all information entered here is included in the Methods section of the manuscript.**	

Powered by Editorial Manager® and ProduXion Manager® from Aries Systems Corporation

**Format for specific study types**<br><br>**Human Subject Research (involving human participants and/or tissue)**<br>• Give the name of the institutional review board or ethics committee that approved the study<br>• Include the approval number and/or a statement indicating approval of this research<br>• Indicate the form of consent obtained (written/oral) or the reason that consent was not obtained (e.g. the data were analyzed anonymously)<br><br>**Animal Research (involving vertebrate animals, embryos or tissues)**<br>• Provide the name of the Institutional Animal Care and Use Committee (IACUC) or other relevant ethics board that reviewed the study protocol, and indicate whether they approved this research or granted a formal waiver of ethical approval<br>• Include an approval number if one was obtained<br>• If the study involved *non-human primates*, add *additional details* about animal welfare and steps taken to ameliorate suffering<br>• If anesthesia, euthanasia, or any kind of animal sacrifice is part of the study, include briefly which substances and/or methods were applied<br><br>**Field Research**<br><br>Include the following details if this study involves the collection of plant, animal, or other materials from a natural setting:<br>• Field permit number<br>• Name of the institution or relevant body that granted permission	
**Data Availability**<br><br>Authors are required to make all data underlying the findings described fully available, without restriction, and from the time of publication. PLOS allows rare exceptions to address legal and ethical concerns. See the PLOS Data Policy and FAQ for detailed information.	Yes - all data are fully available without restriction

A Data Availability Statement describing where the data can be found is required at submission. Your answers to this question constitute the Data Availability Statement and **will be published in the article**, if accepted.<br><br>**Important:** Stating 'data available on request from the author' is not sufficient. If your data are only available upon request, select 'No' for the first question and explain your exceptional situation in the text box.<br><br>Do the authors confirm that all data underlying the findings described in their manuscript are fully available without restriction?	
**Describe where the data may be found in full sentences. If you are copying our sample text, replace any instances of XXX with the appropriate details.**<br><br>• If the data are **held or will be held in a public repository**, include URLs, accession numbers or DOIs. If this information will only be available after acceptance, indicate this by ticking the box below. For example: *All XXX files are available from the XXX database (accession number(s) XXX, XXX.)*.<br>• If the data are all contained **within the manuscript and/or Supporting Information files**, enter the following: *All relevant data are within the manuscript and its Supporting Information files.*<br>• If neither of these applies but you are able to provide **details of access elsewhere**, with or without limitations, please do so. For example:<br><br>*Data cannot be shared publicly because of [XXX]. Data are available from the XXX Institutional Data Access / Ethics Committee (contact via XXX) for researchers who meet the criteria for access to confidential data.*<br><br>*The data underlying the results presented in the study are available from (include the name of the third party*	Rodde, Solene; Arsevska, Elena, 2024, "Data and code for analysis of a bi-mode network of sources and outbreaks of Avian influenza and African swine fever, identified by the PADI-web media monitoring tool", https://doi.org/10.18167/DVN1/W858OB, CIRAD Dataverse, V1, UNF:6:znQlsk3wqBA6ltVQzBaE3g== [fileUNF]

Powered by Editorial Manager® and ProduXion Manager® from Aries Systems Corporation

and contact information or URL).<br>• This text is appropriate if the data are owned by a third party and authors do not have permission to share the data.<br><br>* typeset	
Additional data availability information:	Tick here if the URLs/accession numbers/DOIs will be available only after acceptance of the manuscript for publication so that we can ensure their inclusion before publication.

Powered by Editorial Manager® and ProduXion Manager® from Aries Systems Corporation

Manuscript

Click here to access/download;Manuscript;PLOS_PADI_bipartite_ERGM.pdf

Modelling the drivers of outbreak communication from event-based surveillance for improved epidemic intelligence

Solene Rodde[1,2], Pachka Hammami[1,2◑], Asma Mesdour[1,2], Sarah Valentin[1,3], Bahdja Boudoua[3,4], Paolo Tizzani[5], Lina Awada[5], Carlene Trevennec[2, 4], Paulo Pimenta[6], Andrea Apolloni[1,2◑], Elena Arsevska[1,2◑*]

1 French Agricultural Research Centre for International Development (CIRAD), Montpellier, France
2 Joint Research Unit Animals, Health, Territories, Risks, and Ecosystems (UMR ASTRE), French Agricultural Research Centre for International Development (CIRAD), National Research Institute for Agriculture, Food and Environment (INRAE), Montpellier, France
3 Joint Research Unit Land, Environment, Remote Sensing and Spatial Information (UMR TETIS), French Agricultural Research Centre for International Development (CIRAD), National Research Institute for Agriculture, Food and Environment (INRAE), Montpellier, France
4 National Research Institute for Agriculture, Food and Environment (INRAE), Montpellier, France
5 Data Integration Department, World Organisation for Animal Health (WOAH), Paris, France
6 Independent researcher, Montpellier, France

◑These authors contributed equally to this work.
* Correpondence: elena.arsevska@cirad.fr

Abstract

Epidemic intelligence (EI) practitioners at health agencies monitor various media sources to detect and follow up on disease outbreaks. Several tools, such as PADI-web (Platform for Automated Extraction of Disease Information from the Web), automatically monitor and filter outbreak-related news on the web, facilitating the risk assessment by EI practitioners. However, in the mass of collected news, there is a need to understand better what drives the communication on outbreaks by the different sources to allow for a more targeted and efficient EI process.

We built a bipartite network of sources communicating on outbreaks of avian influenza (AI) and African swine fever (ASF) captured by the PADI-web tool between 2018 and 2019 worldwide to assess a range of epidemiological, socio-economic, and cultural factors that drive communication on disease outbreaks. Our AI network comprised 969 communicated news (links) from 436 news reports from 212 sources describing 199 AI outbreaks. The ASF network comprised 1340 communicated news (links) from 594 news reports from 204 sources and 277 ASF outbreaks.

An Exponential Random Graph Model (ERGM) was fitted for each network. In both models, international organisations and press agency sites were more likely to communicate about outbreaks than online news sites (OR=4.8 and OR=3.2, $p<0.001$ for AI; OR=3.1 and OR=4.7, $p<0.001$ for ASF). Research organisations for AI (OR=2.3, $p<0.001$) and veterinary authorities for ASF (OR=3.6, $p<0.001$) were also more likely to be a source of information than online news sites.

Our work provides insight into the factors driving online communication about animal and zoonotic infectious disease outbreaks. This information can guide EI practitioners to target specific sources based on their specialisation and global coverage and focus on certain world regions based on the disease's epidemiological status.

Introduction

The recent expansion of Internet access and usage has provided a new way to communicate on health events, including disease outbreaks, information that can be used by public and veterinary health (PH/VH) agencies for epidemic intelligence (EI) purposes. Until recently, the EI was based on the assessment of outbreak information collected through the formal process of disease observation by a (veterinary) health practitioner, laboratory confirmation of the suspected pathogen, and PH/VH agency notification to international organisations, such as the World Organisation for Animal Health (WOAH), in the case of mandatory notifiable diseases. Consequently, this process could take days to months, depending on the country's capacities, both material (e.g., equipment and diagnostic tests available) and personnel (e.g., staff available).

Therefore, in recent years, EI practitioners have been increasingly monitoring online sources to obtain timely information on outbreaks, sources ranging from ministerial websites, mainstream and specialised news sites to publicly available databases for disease outbreak notification [1–3]. However, with the enormous number of sources monitored and EI information gathered [4], there is a need to understand better the relation between the disease outbreaks and the sources that communicate on outbreaks so that there is a more targeted EI. A more targeted EI could guide EI practitioners in monitoring, for example, specific sources of information in specific regions of the world where outbreaks occur (REF).

A recent study [5] reconstructed the network of information pathways in the news about avian influenza outbreaks worldwide. The outbreak information pathways comprised the primary source, i.e., the earliest emitter source on a given outbreak mentioned in the news, the intermediary sources that took over the information from the primary source but further communicated the news until the final aggregator, the online news site. The authors showed that the local veterinary authorities were often the primary sources, while WOAH was an intermediary source of outbreak information communicated by the online news sites.

We built upon our previous work [5] and used a statistical modelling approach for network analysis to assess what drives the communication of outbreak information for animal and zoonotic infectious diseases, given different epidemiological, socio-economical and cultural factors associated with the country of the outbreaks and types of sources.

Materials and methods

Case studies

We have focused our work on two diseases known to be a global burden from a health and economic perspective: avian influenza and African swine fever. These are two highly contagious transboundary animal infectious diseases, and avian influenza is also zoonotic.

Avian influenza (AI) is a viral and zoonotic infectious disease affecting many bird species and mammals. Primary hosts are domestic and wild aquatic birds, including poultry, ducks and turkeys, causing significant economic losses in the poultry industry. The disease's main reservoirs are wild aquatic birds (ducks, geese, seagulls, etc.). Transmission occurs mainly by direct contact with infected birds or indirectly through

the environment through excrement and respiratory secretions [6]. In addition, this viral disease presents a public health threat. The AI virus can easily cross the species barrier and infect mammals, including humans. The number of AI infections in fowls surged dramatically between 2018 and the first half of 2022, surpassing the numbers of all preceding years. Human AI infections increased over 2018, 2019, and 2020; these infections were mainly reported in China in 2021 and the first part of 2022. More recently, aquatic mammals and other terrestrial species have been involved in the AI outbreaks [7–9].

African swine fever (ASF) is a viral hemorrhagic disease of domestic and wild pigs. The infection causes massive animal losses and poses a major threat to pig production [10]. The virus is highly contagious and highly resistant in the environment. The disease is endemic in most South-Saharan African countries, while in Europe, outbreaks have been declared annually since its first introduction in Eastern Europe in 2007. The virus has also spread to several Asian countries, where it was detected in farms in China (People's Republic of) in 2018 and then in Oceania in 2019. More recently, the virus has been detected in America after 40 years of absence, with a large spread in the Dominican Republic and Haiti in 2021 [11].

Data

PADI-web. We used the PADI-web tool (Platform for Automated extraction of animal Disease Information from the web) to collect online news data. This tool was developed in 2016 for the International Health Monitoring Unit of the French Platform for Epidemic Surveillance in Animal Health (ESA) to complement the EI activities through monitoring outbreak information communicated in Google News [1, 4]. The tool screens Google News, with search queries ranging from disease names (specific disease surveillance in the news) to combinations between symptoms and hosts (syndromic surveillance in the news). The tool uses an automatic text mining approach to identify and extract epidemiological information on the health event communicated in the news, including the location and date, symptoms, hosts, and number of cases. The tool also keeps track of the news website and publication date.

News reports. For this work, we used the Google News reports collected by PADI-web between July 1, 2018, and June 30, 2019, that communicated one or more AI and ASF outbreaks (436 news reports for AI and 594 for ASF, respectively). These news reports were collected using the search queries "Avian influenza" and "African swine fever" in English. We selected a one-year study period to capture the spatiotemporal epidemiological aspects of the two disease epidemics worldwide, thus allowing us to have a more complete overview and interpretation of the modelling results further implemented on the dataset.

Outbreaks. We further created a database of all outbreak-related events that were communicated in the news that also held information on the date of occurrence, location and host. Each outbreak-related event communicated in the news was further linked to the corresponding officially reported outbreak by the country's competent authorities to the WOAH (199 matched outbreaks for AI and 277 for ASF). Unmatched outbreaks, i.e. outbreaks from the news that could not be linked to the WOAH outbreaks, were discarded from the analysis as we could not verify their truthfulness (31 outbreaks for AI and 69 for ASF).

Sources. The sources were identified manually by tracing all sources of outbreak information mentioned in the news (e.g. Reuters [source]: "Based on WOAH [source], an AI outbreak in a large poultry farm was declared yesterday. On a statement from the Ministry of Agriculture [source] given today, over thousands of poultry were culled"), including the news site itself (e.g., "Reuters"). We further created a link

between each source identified and the outbreak they described [5]. We identified 212 unique sources for AI and 204 unique sources for ASF.

Covariates. For the modelling, we used two groups of covariates [5,12,13]; the first group was related to the sources (n=3) and the second to the outbreaks (n=13).

- Source covariates were:
  - Type: international organisation (e.g., WOAH), company/association (e.g., National Pig Association), laboratory (e.g., Bulgarian National Reference Laboratory), official authority (e.g., customs), veterinary authority (e.g., Ministry of Agriculture), online news (e.g., The New York Post), press agency (e.g., Reuters), radio/TV (e.g., Belstav.tv), social platform (e.g., Twitter), research organisation (e.g., CIDRAP);
  - Geographic focus (international, national, local);
  - Specialisation (general vs specialised health-oriented).
- Outbreak covariates were related to:
  - The epidemiological context with the:
    1. Surveillance capacity of the country, an indicator based on a country's self-assessment informing its capacity to detect, assess, notify, report and respond to public health risks and acute outbreaks of national and international concern (ranging from 0 to 100) [14];
    2. Reason for notification to WOAH: first occurrence in a zone/country, recurrence of the disease, new strain and unknown reason, in case there was no information available [15];
    3. Type of host (domestic vs wild). The domestic pig and poultry were considered domestic; the remaining were considered wild or environmental if the sample came from the environment;
    4. Poultry and pork/swine export, respectively (in US$; total value of the animals exported by a country in a given year) [16].
  - Socio-economical context at the country level and given year, with
    1. Political stability, an estimate measuring perceptions of the likelihood of political instability and politically motivated violence, including terrorism, (ranging from -2.5 to 2.5; lower the value, lower the stability) [17];
    2. Corruption control, an estimate of the control of corruption (ranging from -2.5 to 2.5; lower the value, lower the control) [17];
    3. Gross Domestic Product (GDP) per capita [17];
    4. Gross National Income (GNI) per capita [17];
    5. Human Development Index (HDI) [18];
    6. The internet usage, considered using the percentage of the population using the internet [17].
  - The cultural context at the country level, with:
    1. English-speaking country ('yes' if English is an official language, or 'no');
    2. Press Freedom Indicator, an indicator developed by RSF (Reporter Sans Frontière) which measures the degree of press freedom enjoyed by journalists and media in a country on a scale of 0 to 100 [19];
    3. World region (African Region [AFR], Region of the Americas [AMR], Southeast Asian Region [SEAR], European Region (EUR], Eastern Mediterranean Region [EMR], Western Pacific Region [WPR]) [20].

All covariates associated with the outbreaks were collected and standardised at the country level, except for the reason of notification and type of host, which corresponded to each outbreak.

Colinearity of covariates. The potential correlation between the continuous quantitative covariates was assessed using a Pearson correlation test. Only variables with a correlation coefficient between -0.7 and 0.7 were retained for further analysis. For AI, the Pearson correlation test revealed a significant correlation ($r > 0.70$; $p < 0.05$) between poultry export, GNI per capita and GDP per capita; we decided to keep the GDP per capita covariate as the sole indicator because it is commonly used to assess a country's economic situation and overall economic activity. Pearson correlation test also revealed a significant correlation between the corruption control and political stability variables ($r > 0.70$; $p < 0.05$). The political stability variable was retained because it can be considered an overall indicator considering various factors such as governance, political instability, and violence. Political stability, therefore, reflects not only corruption control but other relevant estimates that contribute to the stability of a country.

For ASF, the Pearson correlation test revealed a significant correlation ($r > 0.70$; $p < 0.05$) between pork export and surveillance - we kept the surveillance covariate. We kept the GDP per capita between corruption control, GDP per capita and GNI per capita. There was also a correlation between the corruption control estimate, internet usage, and HDI; we kept the internet usage covariate since it was also a variable in the AI outbreaks dataset, thus allowing for a better comparison of results between the two diseases.

Network modelling

To best model the interactions between the sources and outbreaks, we created a bipartite (two-mode) network with two nodes of different nature: nodes for sources and nodes for outbreaks (Fig. 1) for each disease, respectively. The links between the two modes corresponded to the communicated news on an outbreak from a given source. In this type of network, links only connect nodes of different types: sources and outbreaks [21]. Covariates were implemented in the network as node attributes.

Exploratory analysis. For each network, a preliminary exploratory analysis was implemented as follows:

- **Global level**. We assessed the network density, i.e., the ratio of the number of edges to the total number of possible edges. We also checked for the network components (weakly vs. strongly connected components). Components are portions of the network that are disconnected from each other. An isolated component is a subset of nodes that are not connected to other nodes in the network.

- **Node level**. We calculated the degree of each node in the network (for sources and outbreaks, respectively), i.e., the number of connections it has to other nodes. The degree distribution was the probability distribution of these degrees over the whole network. More precisely, the source degree showed the number of times a given source communicated about an outbreak, while the degree of an outbreak was the number of times a given outbreak was communicated in the news.

Exponential random graph model. We used a bipartite exponential random graph model (ERGM) to identify the factors that influence the creation of links between a source and an outbreak and, more precisely, to assess what drives a source to communicate on an outbreak-related event. ERGM is a class of statistical models for

network analysis and simulation. In our case, it aims to analyse the characteristics of its nodes and their links to assess news communication on outbreaks. Based on these characteristics, ERGMs simulate networks, identifying the probability distribution of any network [22]. We have chosen ERGM since they have proven useful in modelling social networks and health topics [7, 23, 24].

The model parameters were selected by sequential tests, following a simple stepwise procedure to identify the most parsimonious parameter combination, as described in Hammami et al., 2022 [24]. Starting with a prospective approach from the simplest model, including only links as a predictor, the addition of each network statistic was tested sequentially to identify a relevant subset of predictors (network statistics) based on the Akaike Information Criterion (AIC) indicator. Then, a retrospective approach was used, sequentially removing previously selected predictors from the model to retain only those improving model fit. Forward and backward approaches were implemented until the AIC, indicator of model parsimony, could not be improved by adding or removing a variable. Model fits were only considered when ERGM fitting stopped after the first estimate (using maximum pseudo-likelihood estimates, MPLE) or when maximum likelihood estimation (MLE) converged without generating a warning (using a Markov Chain Monte Carlo (MCMC) estimation algorithm with a Metropolis-Hastings sampler.

Model validation. In-sample performance was assessed for the ERGM selected for each disease by a goodness-of-fit (Gof) analysis comparing three structural statistics of the observed network to those of networks randomly simulated by the adjusted ERGM (minimum geodesic distance, distribution of degree per type of node) [25]. One thousand networks were randomly simulated to achieve the Gof for the network models. The statistics compared were the degree of source nodes, degree of outbreak nodes and minimum geodesic distance.

Software. The analysis was carried out using the R programming language (version 4.3.1) [26], and the associated packages: "igraph" (version 2.0.1.1) [27], "network" (version 1.18.2) [28], "ergm" (version 4.6.0) [29] and "bipartite" (version 2.19) [30].

Ethical approval was not required for this study as it includes information freely available in the public domain. This research does not contain any studies with animals or humans performed by any of the authors.

Results

Exploratory analysis

Avian influenza

The AI network comprised 212 sources, 199 outbreaks, and 969 links (Fig. 2, Fig. 3, Supp file 1). The network had low density (0.023), indicating low node connectivity. The network had four components, with 396 nodes in the principal component and 8, 4, and 3 nodes in the remaining components, respectively, indicating low connectivity between nodes. The most important sources were the WOAH (deg = 140), Center for Infectious Disease Research and Policy (CIDRAP) (deg = 129) and WATTAgNet online news (deg = 59). The highest degree concerned three outbreaks in India (deg = 36, deg = 22, deg = 20, respectively), followed by outbreaks occurring in the United States of America (deg = 20) and Malaysia (deg = 19) (Supp file 2).

African swine fever

The ASF network comprised 204 sources, 277 outbreaks, and 1340 links (Fig. 2, Fig. 3, Supp file 1). The network had low density (0.024) and four components (467 nodes in

the principal component and 3, 8, and 3 nodes in the remaining components, respectively), indicating low connectivity between nodes. The most important sources were the Reuters press agency (deg = 158), China Veterinary Authority (deg = 125), and the Xinhua press agency (deg = 87). Among the five outbreaks with the highest degrees, one occurred in China (deg = 24), one in North Korea (deg = 21), one in Slovakia (deg = 16) and two in Belgium (deg = 15, deg = 14, respectively) (Supp file 2).

Exponential random graph model

Table 1 shows the key factors for information dissemination on AI and ASF outbreaks selected using the stepwise procedure.

**Type node**	**ERGM terms**	**Covariate**	**AI**	**ASF**
Source	nodefactor	Type source	X	X
		Geographical focus	X	X
		Specialisation	X	
Outbreak	nodefactor	Type host	X	
		Political stability	X	
		GDP per capita	X	X
		Internet usage	X	X
		Official language	X	
		Region	X	X
		PFI	X	X
	nodematch	Region (homophily)	X	

Table 1. Covariates retained (shown as X) with a stepwise selection procedure for Avian influenza (AI) and African swine fever (ASF)

Avian influenza. The AI model was obtained with a stepwise selection procedure comprised of eleven covariates (Table 1, AIC of 7175). Based on the odds ratio, the most important covariates involved in the likelihood of a link between sources and AI outbreaks were the source type, the geographical focus, the specialisation of the source, and the region and language of the country where the AI outbreaks occurred (Fig. 4).

Type of source. The international organisations, research organisations and press agencies had the highest probability of forming links with outbreaks compared to online news (OR=4.82, OR=2.29, OR=3.17, $p<0.001$, respectively; Fig. 4). Social platforms and veterinary authorities were less likely to communicate about AI outbreaks ($p<0.001$; Fig. 4).

Geographical focus and specialisation of the source. International and national coverage sources were more likely to communicate about outbreaks of AI than local sources (OR=2.99, OR=1.95, $p<0.001$, respectively; Fig. 4). Specialised sources were 4.4 times more likely to communicate information on AI outbreaks than generalised sources ($p<0.001$; Fig. 4).

Region of the outbreaks. AI outbreaks in Europe, America and Southeast Asia were more likely to be communicated by sources (OR=2.57, OR=9.95, OR=2.89, $p<0.001$, $p=0.002$, $p<0.001$, respectively; Fig. 4).

Host type. AI outbreaks in wild birds were more likely to be communicated by the sources than outbreaks affecting domestic birds (OR=1.67, $p<0.001$; Fig. 4).

Official language. AI outbreaks occurring in a country where English is the official language were more likely to be broadcast by sources than in non-English-speaking countries (OR=1.76, p<0.001; Fig. 4).

African swine fever. The ASF model obtained with a stepwise selection process comprised six covariates (Table 1, AIC of 11789), with the most impacting covariates being the type and geographical focus of the source and the region of the outbreaks (Fig. 5).

Type of source. Similarly to the AI model, sources such as international organisations and press agencies were more likely to form links with outbreaks compared to online news sources (OR=3.08, OR=4.71, respectively, p<0.001; Fig. 5). However, compared to the AI model, the veterinary authorities were more likely to communicate about ASF outbreaks compared to online news sources (OR=3.64, p<0.001; Fig. 5).

Geographical focus of the source. Similarly to the AI model, international and national coverage sources were more likely to communicate about outbreaks of ASF than local sources (OR=8.60, OR=5.29, p<0.001, respectively; Fig. 5).

The Gof statistics showed a good model fit for both the AI and ASF models (Supp file 3), with a slightly better fit for the AI model.

Discussion

This study assessed the factors influencing the communication of AI and ASF outbreaks in online news sources using ERGMs applied to a bipartite network of sources and outbreaks. Our results show that the main factors influencing information dissemination on AI and ASF outbreaks are the source type, geographical focus, and the region where the outbreaks occur. In addition, for the AI model, communication regarding outbreaks depends on the specialisation of the sources, the type of host, and the language spoken in the country where the outbreaks occurred.

Role of the source type and specialisation in outbreak communication. Our results showed that WOAH was the major source of outbreak information, particularly for AI. This is not a surprise since previous works showed that in the news, WOAH is frequently cited as an information source [5]. These results suggest that online media sources frequently check for WOAH-reported outbreaks, which are available publicly online through the World Animal Health Information System (WAHIS). Both the WAHIS website and WAHIS distribution list facilitate the dissemination and the use of health information by web sources for situation information and awareness on trending epidemics and pandemics [5, 31]. Therefore, EI practitioners should be aware, when monitoring online media sources, that much of the information in the news may originate from WOAH.

Similarly, press agencies were more likely to communicate about disease outbreaks for AI and ASF. These sources represented 6% and 7% out of the total number of sources for AI and ASF, respectively. Some press agencies with worldwide coverage, such as Reuters, were also in our database and among the top three sources reporting ASF outbreaks. These agencies have newsagents or reporters who provide news and updates often directly from the field, thus probably explaining the higher likelihood of communicating on disease outbreaks. As shown in previous works, mass media sites were the major sources of information about the COVID-19 pandemic, much like the previous pandemics of SARS (2003), H1N1 (2009), and MERS (2012) [32, 33].

Finally, research organisations, notably CIDRAP, were also important in communicating about outbreaks, particularly for AI. At the same time, veterinary authorities were frequently a source of information on ASF outbreaks. These results show that conducting research is not solely about contributing to the evidence base; communicating research findings is equally important to achieve an effective public health response and awareness [7]. In the context of ASF, the risk communication of veterinary services seemed important in providing information and awareness for ASF prevention and control, which was also noted in the online media news. Since ASF is a highly contagious infectious disease with no cure and no vaccination options, effective risk communication and awareness are critical to preventing its spread, as reflected in the news reports we analysed, where veterinary authorities were a source of ASF outbreak information.

Role of the language and region of the outbreaks. Our results showed that outbreaks in English-speaking countries were more likely to be communicated than [illegible]e in non-English-speaking countries, particularly for AI. Epidemics and disease outbreaks can be fast-spreading, so risk communication needs to keep up. To deliver timely information, we hypothesise that news sources tend to (re-)use the information published or available in English rather than in a local language to avoid low-quality translation and non-timely communication.

Furthermore, we found that the region where the outbreak occurred plays an important role in its communication, particularly for AI. Outbreaks of AI occurring in America, Europe, and Southeast Asia were more likely to be communicated in the news. [illegible]s may be related to the extension and gravity of the epidemic in these regions in 2018/19. According to the WOAH database, there were 350 outbreaks of AI officially notified by national authorities in 30 countries and 5205 outbreaks of ASF during this period in 21 countries worldwide. The majority of AI outbreaks were notified in Chinese Taipei (89; 25.4%), followed by 56 (16%) in Russia and 27 (7.7%) in India. The majority of the ASF outbreaks were notified in Poland (2024; 38.9%), followed by 1334 (25.6%) in Romania and 658 (12.6%) in Belgium.

Limitations and perspectives.

While ERGMs have been widely used for monopartite networks in health and disease research [23, 34], their use on bipartite networks is relatively recent, and it seems necessary to develop other methods for analysing bipartite networks. Our model results were based on two networks constructed based on online news reports and outbreaks in 2018/19. However, to confirm our model outcomes, we envisage enriching the dataset by covering a larger period beyond one year.

Our work presents the results of two global models without considering the time, i.e., the dates of the outbreak communication. In our future work, we plan to consider the time component. One way of doing that is to divide the global network into two sub-networks based on a given source's timeliness, i.e., whether a source communicated about an outbreak before or after the date of official notification to WOAH [35]. This may allow us to assess if different factors are at stake in the early phase of an epidemic, where the news aims to alert on outbreaks, compared to a stage when the epidemic is established where the news provides a follow-up and synthesis, rather than breaking news [36].

In addition, a major point that needs to be explored further is the role of the sources at different levels, such as the news sources captured by PADI-web (e.g., Reuters) vs. the cited sources in the news itself (e.g., WOAH, Ministry of Agriculture) [5]. This information about whether a source is a ”final source” or a ”cited source” is an attribute of the link, not the node. While it is possible in ERGM on a monopartite network to integrate link attributes into the model, to our knowledge, it hasn't yet been implemented for bipartite networks and needs further exploration.

The news reports used in this work were obtained based on search queries in English. Thus, PADI-web captured Google News in English, regardless of the country of the news. Therefore, news reports from non-English-speaking countries might have been under-sampled with this approach, thus contributing to a poor generalisation of our results. Therefore, future works should assess a larger corpus of news reports from queries in other languages, as these multilingual queries have been recently added to PADI-web.

Finally, we found that 13% for AI and 20% for ASF outbreaks described in the news reports could not be matched to any event in the WOAH database. Complementing the gold standard from WOAH with other verified outbreak information, such as expert networks, such as ProMED-mail [37–39] could be envisaged in the future to avoid the loss of a potentially valuable information for EI purposes.

Conclusion

Our work provides insight into the factors driving online communication on animal and zoonotic infectious disease outbreaks, given the different epidemiological, socioeconomic, and cultural factors associated with the country of outbreaks and the sources of information. The results of the two disease models, AI and ASF, are very similar. Our results show that practitioners should consider the source type when conducting intelligence activities, ranging from international organisations and authorities to press agency websites with global coverage. The outbreak news is most likely related to the endemic/epidemic status in the given region and type of host, and these factors should be considered during the EI.

Supporting information

S1 File. Bipartite network of sources communicating on Avian Influenza and African swine fever between outbreaks 2018 and 2019 extracted with the PADI-web tool.

S2 File. Degree distribution for the node sources and node outbreaks of the bipartite networks for Avian influenza and African swine fever.

S3 File. A. Goodness-of-fit (Gof) analysis for the Exponential random graph Models (ERGM) Avian influenza and African swine fever.

Code and Data Availability

The code and data needed to reproduce the results of this work are available at CIRAD Dataverse: `https://doi.org/10.18167/DVN1/W858OB`.

Author contribution

Conceived and designed the analysis: SV, AM, PH, AA, EA; Collected and curated the data: SR, SV, BB, EA; Performed the analysis: SR, PP; Wrote the draft paper: SR, EA; Critical review: SV, LA, PT, CT, AM, PH, PP.

Funding

This study was partially funded by the European Commission (EC) Grant number 874850 - MOOD project, which is catalogued as MOOD 113. This study was also funded by the Direction générale de l'alimentation (DGAL), France.

Ethical statement

Ethical approval was not required for this study as it includes information (news) freely available in the public domain. This research does not contain any studies with animals or humans performed by any of the authors.

Disclaimer

The views and opinions expressed in this article are those of the authors and do not necessarily reflect the official policy or position of the World Organisation for Animal Health (WOAH), the European Commission (EC), or the Direction générale de l'alimentation (DGAL), France.

Conflict of interest

None declared.

References

1. Arsevska E, Valentin S, Rabatel J, Hervé JdGd, Falala S, Lancelot R, et al. Web monitoring of emerging animal infectious diseases integrated in the French Animal Health Epidemic Intelligence System. PLOS ONE. 2018;13(8):e0199960. doi:10.1371/journal.pone.0199960.

2. Freifeld CC, Mandl KD, Reis BY, Brownstein JS. HealthMap: Global Infectious Disease Monitoring through Automated Classification and Visualization of Internet Media Reports. Journal of the American Medical Informatics Association. 2008;15(2):150–157. doi:10.1197/jamia.M2544.

3. Yu VL, Madoff LC. ProMED-mail: An Early Warning System for Emerging Diseases. Clinical Infectious Diseases. 2004;39(2):227–232. doi:10.1086/422003.

4. Valentin S, Arsevska E, Falala S, de Goër J, Lancelot R, Mercier A, et al. PADI-web: A multilingual event-based surveillance system for monitoring animal infectious diseases. Computers and Electronics in Agriculture. 2020;169:105163. doi:10.1016/j.compag.2019.105163.

5. Valentin S, Boudoua B, Sewalk K, Arınık N, Roche M, Lancelot R, et al. Dissemination of information in event-based surveillance, a case study of Avian Influenza. Plos one. 2023;18(9):e0285341.

6. Charostad J, Rukerd MRZ, Mahmoudvand S, Bashash D, Hashemi SMA, Nakhaie M, et al. A comprehensive review of highly pathogenic avian influenza (HPAI) H5N1: An imminent threat at doorstep. Travel Med Infect Dis. 2023;55:102638. doi:10.1016/j.tmaid.2023.102638.

7. Yu F, El-Zaatari HM, Kosorok MR, Carnegie A, Dave G. The application of exponential random graph models to collaboration networks in biomedical and health sciences: a review. Network Modeling Analysis in Health Informatics and Bioinformatics. 2024;13(1):5. doi:10.1007/s13721-023-00439-w.

8. Venkatesan P. Avian influenza spillover into mammals. The Lancet Microbe. 2023;.

9. Sun Y, Zhang T, Zhao X, Qian J, Jiang M, Jia M, et al. High activity levels of avian influenza upwards 2018–2022: A global epidemiological overview of fowl and human infections. One Health. 2023;16:100511. doi:https://doi.org/10.1016/j.onehlt.2023.100511.

10. Penrith ML. Current status of African swine fever. CABI Agriculture and Bioscience. 2020;1(1):11. doi:10.1186/s43170-020-00011-w.

11. Juszkiewicz M, Walczak M, Woźniakowski G, Podgórska K. African Swine Fever: Transmission, Spread, and Control through Biosecurity and Disinfection, Including Polish Trends. Viruses. 2023;15(11):2275. doi:10.3390/v15112275.

12. Ganser I, Thiébaut R, Buckeridge DL. Global Variations in Event-Based Surveillance for Disease Outbreak Detection: Time Series Analysis. JMIR Public Health and Surveillance. 2022;8(10):e36211. doi:10.2196/36211.

13. Oppenheim B, Gallivan M, Madhav NK, Brown N, Serhiyenko V, Wolfe ND, et al. Assessing global preparedness for the next pandemic: development and application of an Epidemic Preparedness Index. BMJ Glob Health. 2019;4:e001157. doi:10.1136/bmjgh-2018-001157.

14. Organization WH. Outbreak communication: best practices for communicating with the public during an outbreak; 2005. Available from: `https://www.who.int/publications/i/item/outbreak-communication-best-practices-for-communicating-with-the-public-d`

15. WOAH. WAHIS: World Animal Health Information System; 2024. `https://wahis.woah.org/`.

16. FAO. Food and agriculture data; 2024. `https://www.fao.org/faostat/en/#home`.

17. The World Bank W. DataBank; 2024. `https://databank.worldbank.org/`.

18. Roser M. Human Development Index (HDI). Our World in Data. 2014;.

19. RSF. Reporter Sans Frontière, Press Freedom Indicator; 2024. `https://rsf.org/en/index`.

20. WHO. World Health Organization, World health statistics 2010. World Health Organization; 2011.

21. Asratian AS, Denley TMJ, Häggkvist R. Bipartite Graphs and their Applications. Cambridge University Press; 1998.

22. Caimo A, Gollini I. Recent Advances In Exponential Random Graph Modelling. Mathematical Proceedings of the Royal Irish Academy. 2023;123A(1):1–12. doi:10.1353/mpr.2023.0000.

23. Silk MJ, Croft DP, Delahay RJ, Hodgson DJ, Weber N, Boots M, et al. The application of statistical network models in disease research. Methods in Ecology and Evolution. 2017;8(9):1026–1041. doi:10.1111/2041-210X.12770.

24. Hammami P, Widgren S, Grosbois V, Apolloni A, Rose N, Andraud M. Complex network analysis to understand trading partnership in French swine production. PloS one. 2022;17(4):e0266457.

25. Hunter DR, Goodreau SM, Handcock MS. Goodness of Fit of Social Network Models. Journal of the American Statistical Association. 2008;103(481):248–258. doi:10.1198/016214507000000446.

26. R Core Team. R: A Language and Environment for Statistical Computing; 2021. Available from: https://www.R-project.org/.

27. Csardi G, Nepusz T. The Igraph Software Package for Complex Network Research. InterJournal. 2005;Complex Systems:1695.

28. Butts CT. network: A Package for Managing Relational Data in R. Journal of Statistical Software. 2008;24:1–36. doi:10.18637/jss.v024.i02.

29. Hunter DR, Handcock MS, Butts CT, Goodreau SM, Morris M. ergm: A package to fit, simulate and diagnose exponential-family models for networks. Journal of statistical software. 2008;24(3):nihpa54860.

30. Dormann CF, Gruber B, Fründ J. Introducing the bipartite package: analysing ecological networks. interaction. 2008;1(0.2413793):8–11.

31. Lin SY, Beltran-Alcrudo D, Awada L, Hamilton-West C, Lavarello Schettini A, Cáceres P, et al. Analysing WAHIS Animal Health Immediate Notifications to Understand Global Reporting Trends and Measure Early Warning Capacities (2005–2021). Transboundary and Emerging Diseases. 2023;2023:e6666672. doi:10.1155/2023/6666672.

32. Anwar A, Malik M, Raees V, Anwar A. Role of Mass Media and Public Health Communications in the COVID-19 Pandemic. Cureus. 2020;doi:10.7759/cureus.10453.

33. Collinson S, Khan K, Heffernan JM. The Effects of Media Reports on Disease Spread and Important Public Health Measurements. PLOS ONE. 2015;10(11):e0141423. doi:10.1371/journal.pone.0141423.

34. Goeyvaerts N, Santermans E, Potter G, Torneri A, Van Kerckhove K, Willem L, et al. Household members do not contact each other at random: implications for infectious disease modelling. Proceedings of the Royal Society B: Biological Sciences. 2018;285(1893):20182201. doi:10.1098/rspb.2018.2201.

35. Butts CT. Continuous time graph processes with known ERGM equilibria: contextual review, extensions, and synthesis. The Journal of Mathematical Sociology. 2024;48(2):129–171. doi:10.1080/0022250X.2023.2180001.

36. Steele L, Orefuwa E, Dickmann P. Drivers of earlier infectious disease outbreak detection: a systematic literature review. International Journal of Infectious Diseases. 2016;53:15–20. doi:10.1016/j.ijid.2016.10.005.

37. Barboza P, Vaillant L, Strat YL, Hartley DM, Nelson NP, Mawudeku A, et al. Factors Influencing Performance of Internet-Based Biosurveillance Systems Used in Epidemic Intelligence for Early Detection of Infectious Diseases Outbreaks. PLOS ONE. 2014;9(3):e90536. doi:10.1371/journal.pone.0090536.

38. Mercier A, Lancelot R, Hendrikx P, Mackinnon L, Madoff L, Lambert Y, et al. The French Epidemic Intelligence System: comparing disease surveillance at the national and international level using data from the Program for Monitoring Emerging Diseases. Revue Scientifique et Technique de l'OIE. 2020;39(3):805–815. doi:10.20506/rst.39.3.3179.

39. Carrion M, Madoff LC. ProMED-mail: 22 years of digital surveillance of emerging infectious diseases. International Health. 2017;9(3):177–183. doi:10.1093/inthealth/ihx014.

Fig1

Click here to access/download;Figure;fig1_schema_bipartite_network.png

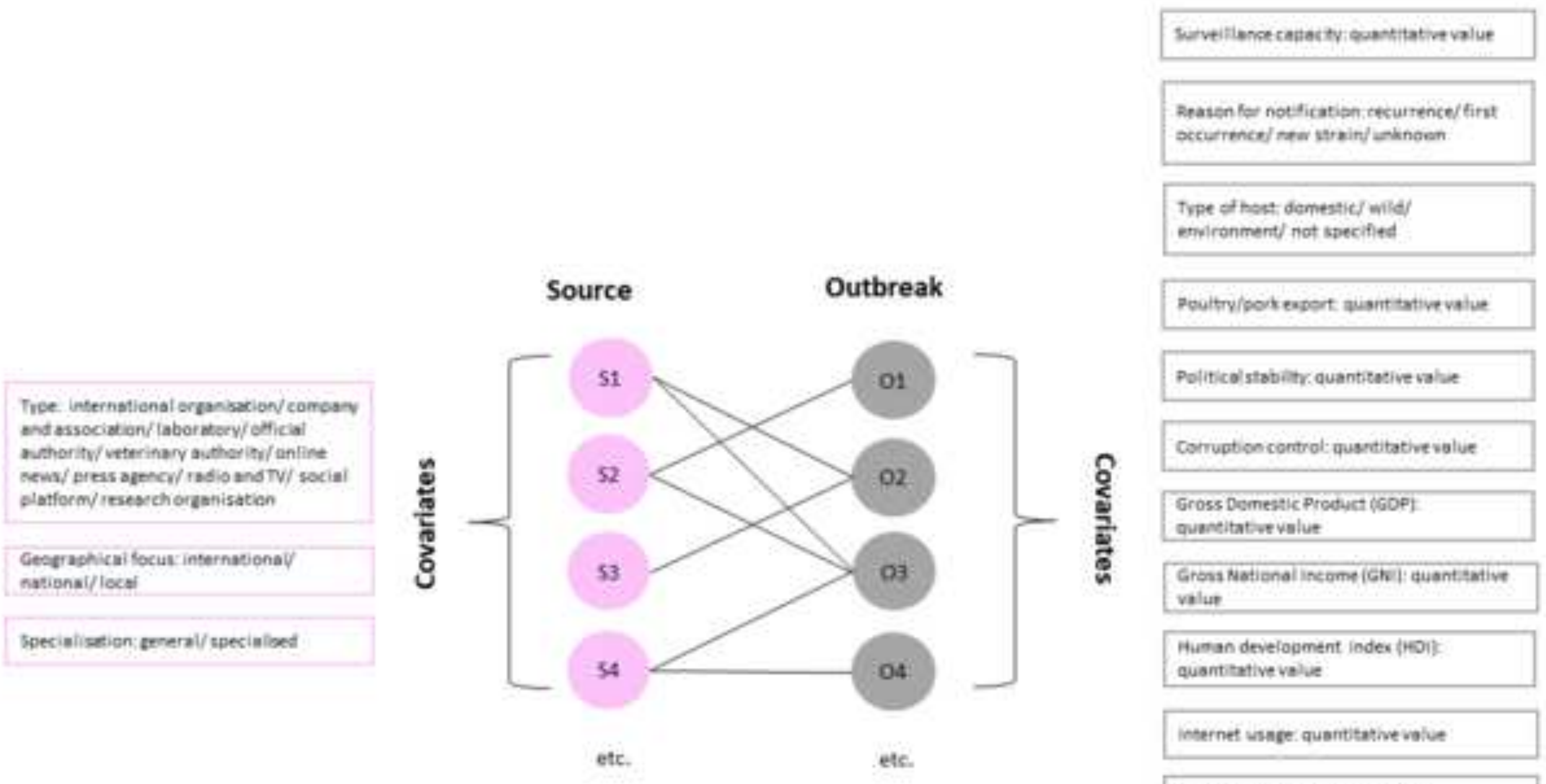

Fig2

Click here to access/download;Figure;fig2_circo_plot_ai_ppa_with_alpha.eps

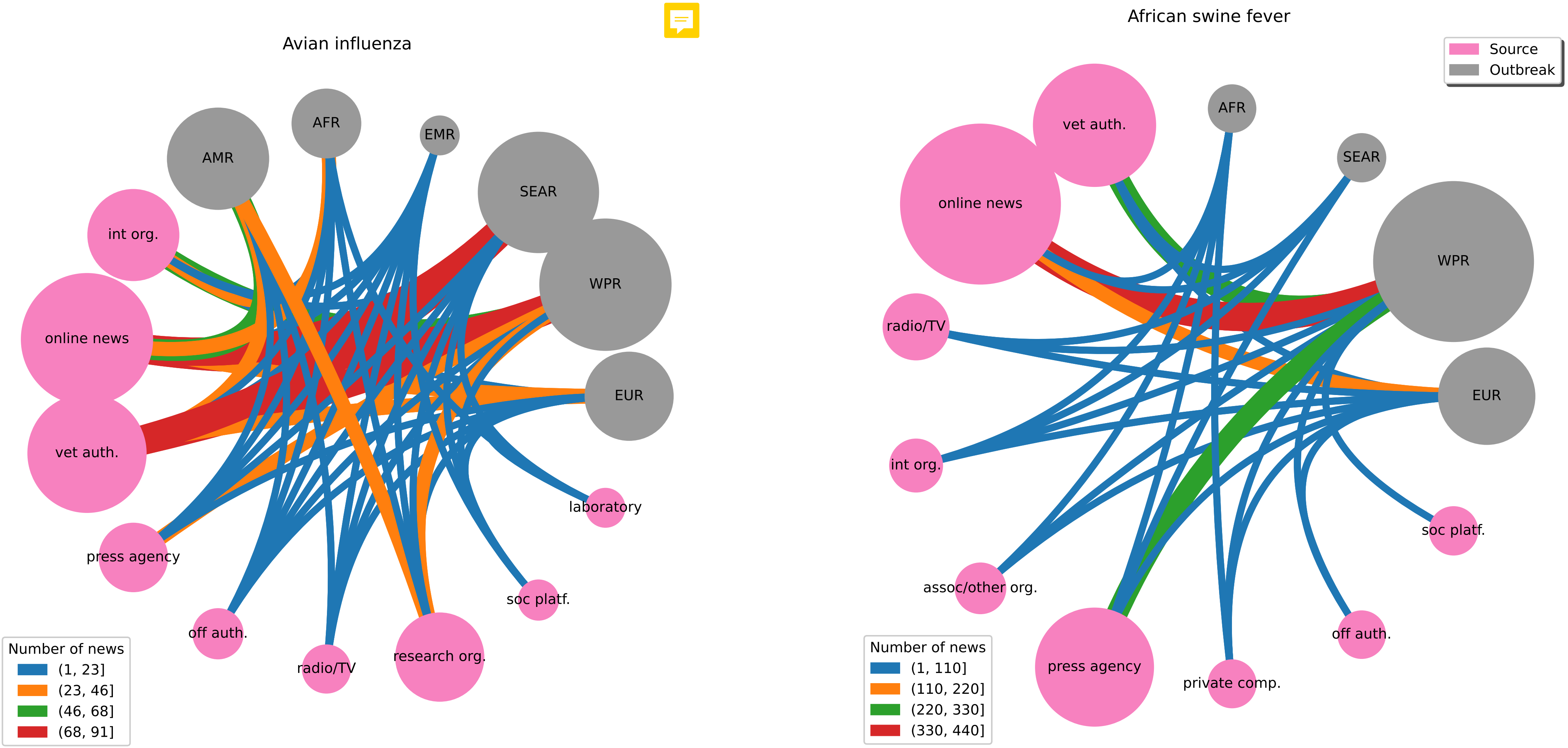

Fig3

Click here to access/download;Figure;fig3_heatmap_ai_ppa.png

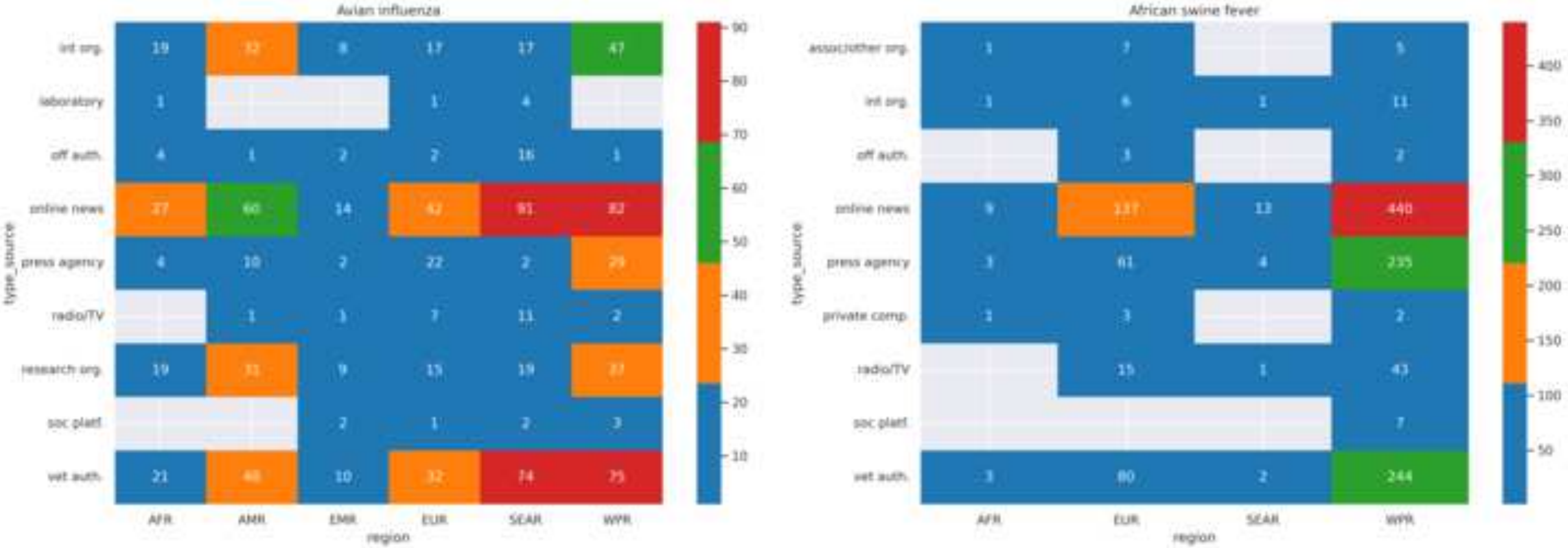

Fig4

Click here to access/download;Figure;fig4_ai_ergm_global.png

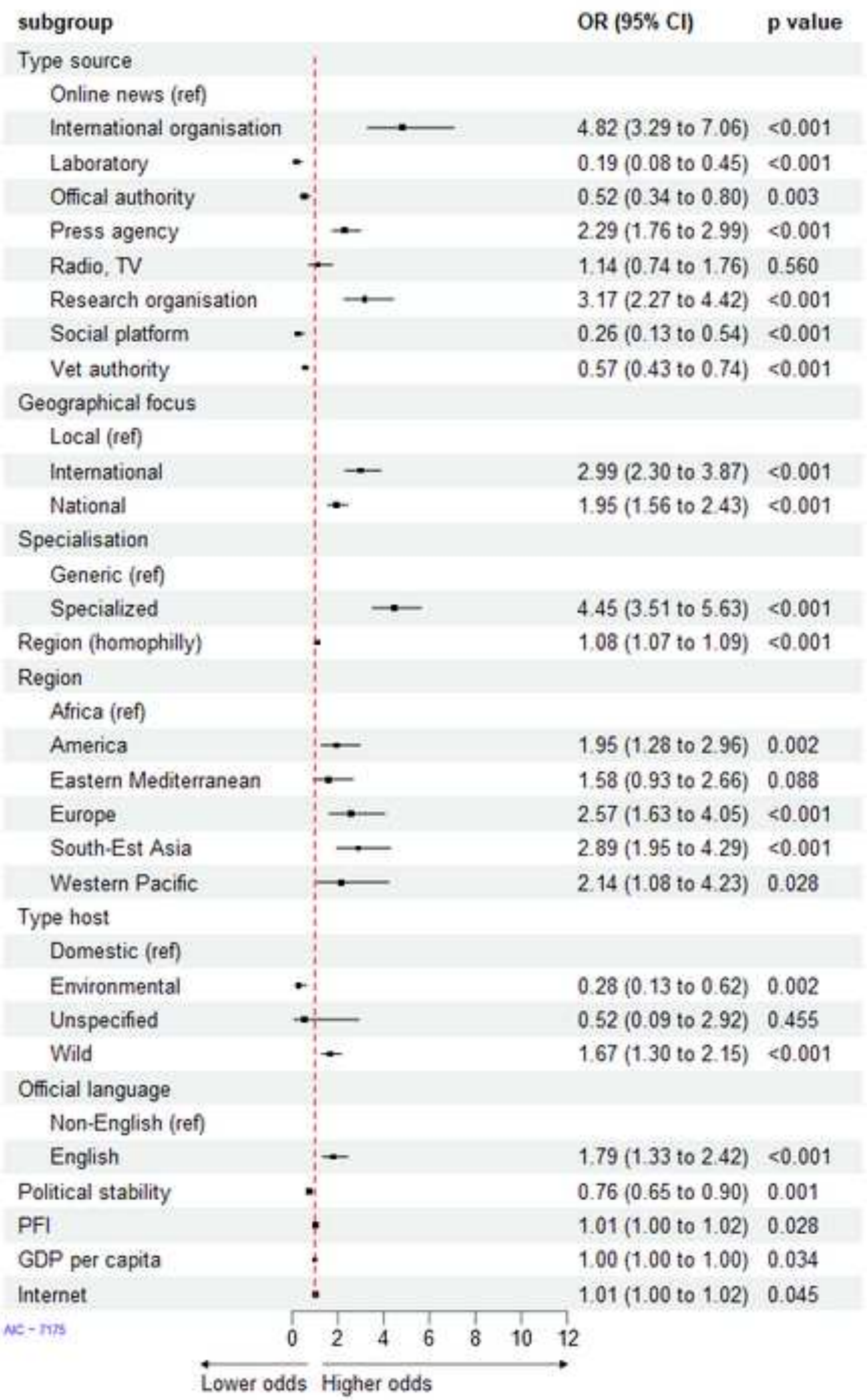

Fig5

Click here to access/download;Figure;fig5_asf_ergm_global.png

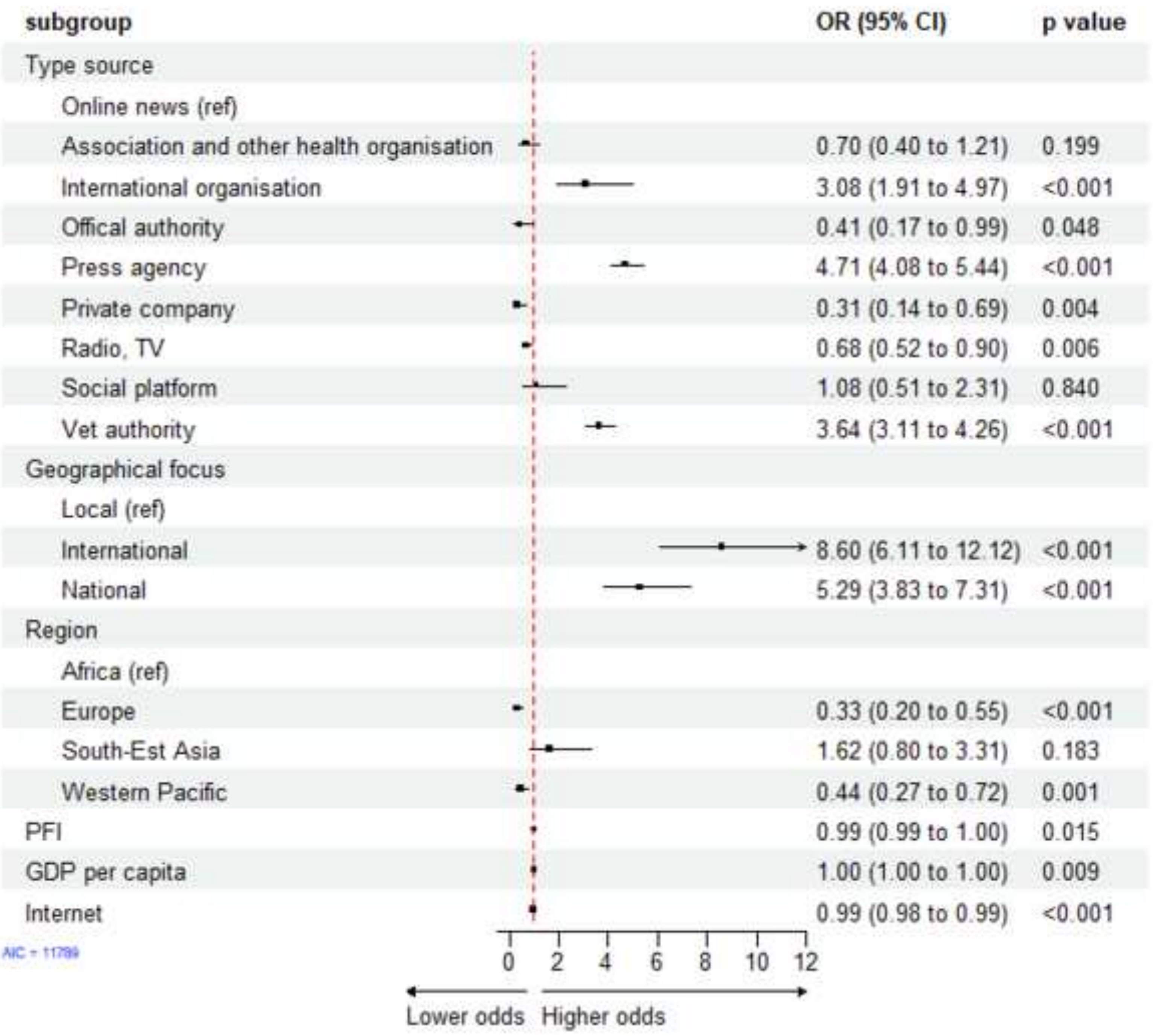

S1_doc

Click here to access/download
Supporting Information
S1_File.docx

S2_doc

Click here to access/download
Supporting Information
S2_File.docx

S3_doc

Click here to access/download
Supporting Information
S3_File.docx
