# Supplementary material for: Modelling the drivers of outbreak communication in online media news for improved event-based surveillance
Source: PLoS One. 2025 Aug 4;20(8):e0327798. doi: 10.1371/journal.pone.0327798 (PMC12321081; doi:10.1371/journal.pone.0327798)
Supplement: Attachment — Submitted filename: Answer to reviewers v2.pdf [file pone.0327798.s005.pdf]

Elena Arsevska, UMR ASTRE, CIRAD, Montpellier, France

Corresponding author: elena.arsevska@cirad.fr

Montpellier 14/04/2025

Manuscript: "Modelling the drivers of outbreak communication in online media news for improved event-based surveillance" (PONE-D-24-25259)

Dear Reviewers,

We provide a revised version of our manuscript, "Modelling the drivers of outbreak communication in online media news for improved event-based surveillance" (PONE-D-24-25259), and the response to your comments and review, outlined below and also reflected in the manuscript (with track changes).

Responses

Overall, I agree with Reviewer 3 that the article clearly describes methods and results and makes no exaggerated claims. However, the discussion and conclusions are still very weak.

We have reworked the entire discussion and the conclusion. In the revised discussion, we focus on four main points based on the primary results but also the comments from the reviewers:

- **The role of online news source types and specialisation for outbreak communication**, with the important role of having reliable, trustworthy sources to avoid panic and misinformation, is something that we saw during the COVID-19 pandemic in the news. The media provides helpful information not just for risk assessors but also for the general public.
- **The timing and dynamics of outbreak communication in the online news** were not our work's objective. Still, we added the results from the preliminary analysis and illustrated examples of how time and dynamics of outbreak communication may be conducted in future works with a larger dataset. We also offer related works on how media monitoring was used by WHO and epidemic intelligence teams in the different stages of COVID-19, even when authorities were aware of the event.
- **Region of the outbreaks and news coverage of online media**, where we better discuss the use of English RSS search feeds to collect data and its biases in not capturing local languages, for example, an outbreak/health event of national/local importance (rather than events of more considerable international concern that we may capture with the English language). We also discuss better the results on the region homophily and why potentially some regions communicate more on outbreaks compared to the African region, which is also related to human and media development, surveillance capacity, and the disease burden, which is multiple in the African countries compared to the other countries from our dataset.
- **The use and comparison of multiple event-based surveillance from various sources for efficient EBS**, not just using online media reports but also local community sources, depending

on the region and local context (some countries in development would use radio/TV and community reports rather than the internet to be informed or inform on health topics).

- **Bipartite network modelling**, with the added value of using this method for bipartite network sources - outbreaks modelling (and its originality in the context of outbreak communication modelling) and perspectives in case we would like to model the dynamics of outbreak communication.

The conclusion was also reworked to represent the main takeaway home messages of our results by epidemic intelligence practitioners.

Reviewer 3 asked, "What is the relative value of EBS systems picking up these reports if health authorities are already aware of the event?" The paper did not adequately answer this question.

This comment is addressed in the subsection “News source types and specialisation for outbreak communication” in the Discussion, lines 262-270.

The added value of media monitoring is to provide EBS capabilities by aggregating data from diverse sources, including reliable, official, and specialised outlets. This enables early intervention and more nuanced risk assessment from EBS sources \cite{togami_2022}, even when health authorities are aware of the event. For example, in the early months of the pandemic, EI from EBS at the WHO complemented official COVID-19 case and death reporting, prompting verification processes for a more timely response. As the pandemic progressed, EI activities at the WHO provided a more comprehensive understanding of the situation, which could not be captured by a single type of surveillance alone \cite{togami_2022}.

Togami et al.: https://www.eurosurveillance.org/content/10.2807/1560-7917.ES.2022.27.49.2200142

In addition, one of the co-authors of our work and daily epidemic practitioner added to the discussion (in the same subsection) an explanation on the use of reliable and official sources from media, but also using media overall as a source (even though she has at her disposal the official WOAH reports). See lines 249-262.

Overall, media surveillance from reliable sources adds significant value to monitoring disease outbreaks and health events, even when epidemic practitioners are already aware of the situation. While official statements from organisations like WOAH WAHIS are critical, data from diverse sources can provide additional insights. By cross-referencing these varied sources, media monitoring helps create a more nuanced and complete understanding of the outbreak's evolution and a more comprehensive risk assessment. Moreover, press agencies play a crucial role in verifying unofficial information, ensuring its reliability before dissemination. For instance, press agencies like Reuters actively evaluate and assign reliability scores to sources, helping ensure that information used in outbreak reporting is trustworthy and accurate. CIDRAP, for example, is now part of the routine epidemic intelligence screening and other sources besides WOAH WAHIS because it provides context and not just timelines.

In addition, there was a question about whether the **dataset was not analysed before and after the official WOAH notification**, and the authors responded to this in the response letter, stating that the dataset was too small to do this. However, this is not reflected in the discussion, and the paper's readers are still left wondering why this was not done. In addition to explaining why this cannot be

done, you should discuss what you can conclude given this shortcoming, considering the question above regarding the value of EBS if the primary sources are official. These two issues are linked.

This comment is addressed in the subsection “Timing and dynamics of outbreak communication in the online news” in the Discussion, lines 271-307.

The question of whether the dataset was analysed before and after the official WOAH notification was raised was addressed in our discussion, explaining that the dataset was too small for a robust analysis of this nature (news on 52 (26%) avian influenza outbreaks and news on 99 (35%) African swine fever outbreaks). However, we provide the results of this analysis, where again, the region of the outbreaks, the source type and specialisation remain essential drivers of early outbreak communication, along with a higher human development index.

We further show some possible useful future works evaluating the timeliness of outbreak news in media and including reporting during different stages of epidemics.

I understand your explanations for why language was not removed from the model. Still, the discussion tries to make sense of language biases as if they were a meaningful result rather than explicitly pointing out that they are an artefact **of the methods.** You state, for example, **"Our results also showed that language and geography may be related".** This is not a result brought to light by this work; this is just a fact. The fact that this information is relevant to the validity of your results and should be considered when interpreting them is crucial for **the discussion.**

Indeed, this part of the discussion was poorly written; thank you for this pertinent comment. We have reworked the subsection “Region of the outbreaks and news coverage of online media”, lines 308-334. We further develop why some reasons may be poorly represented or poorly covered by the online media sources monitored by PADI-web.

Again, we underline that though this current study primarily worked with queries in English, a limitation is that it might have overlooked valuable local sources, often providing more context-specific insights. Understanding this limitation is crucial, as it helps refine future methodologies to incorporate a broader spectrum of local sources, thus enhancing the accuracy and reliability of early warning systems.

An additional subsection, “Event-based surveillance from multiple sources adapted to local context”, lines 335-383, also discusses the importance of using other sources of information and includes local communities where online media may be poorly represented.

A tiny part of the discussion focuses on interpreting the results, and it doesn't leave readers with a clear understanding of the main insights learned and how they can help better implement or utilise **the results of EBS.**
The conclusion paragraph, in particular, provides no specific insight into the take-home message of this paper or the novel knowledge it offers**.** It states, "**We provide the first insight to guide practitioners when monitoring online media for EI purposes." What are the main insights, and how do they guide practitioners?** This point, in particular - how the insights of this paper help practitioners of EBS - is **never really made clear.**

This study explored the factors influencing the communication of AI and ASF outbreaks across online media sources monitored by PADI-web between 2018 and 2019. While based on a single EBS tool and two disease models, our findings show that digital outbreak communication varies between diseases, driven by the disease emergence in specific countries or regions and the involvement of specialised sources, such as national authorities, international organisations, and press agencies. These results highlight the importance of considering both the type and specialisation of sources for EI.

Our analysis also highlights how the region and the language in the country of outbreaks may influence online media coverage. Future research should include a broader range of data sources, region-specific media, and multilingual news coverage to improve the inclusivity and comprehensiveness of EBS, especially in low- and middle-income countries.

EI practitioners should adopt flexible, context-specific strategies for EBS tailored to the unique circumstances of each country or region. Such approaches will ensure a more accurate and thorough understanding of the disease outbreak situation, supporting better-informed and timely risk assessment and decision-making.

In summary, while this study demonstrates what drives outbreak communication in online media, it also highlights the necessity of refining data sources and methodologies to overcome language and regional biases.
